# Silver nanoparticles as a control agent against facades coated by aerial algae—A model study of *Apatococcus lobatus* (green algae)

**DOI:** 10.1371/journal.pone.0183276

**Published:** 2017-08-14

**Authors:** Paulina Nowicka-Krawczyk, Joanna Żelazna-Wieczorek, Tomasz Koźlecki

**Affiliations:** 1 Laboratory of Algology and Mycology, Faculty of Biology and Environmental Protection, University of Łódź, Łódź, Poland; 2 Department of Chemical Engineering, Faculty of Chemistry, Wrocław University of Technology, Wrocław, Poland; VIT University, INDIA

## Abstract

Aerial algae are an important biological factor causing the biodegradation of building materials and facades. Conservation procedures aimed at the protection of historic and utility materials must be properly designed to avoid an increase of the degradation rate. The aim of the present study was to investigate the effect of silver nanoparticles (AgNP) synthetized with features contributing to the accessibility and toxicity (spherical shape, small size) on the most frequently occurring species of green algae in aerial biofilms and thus, the most common biodegradation factor–*Apatococcus lobatus*. Changes in the chloroplasts structure and the photosynthetic activity of the cells under AgNP exposure were made using confocal laser microscopy and digital image analysis and the estimation of growth inhibition rate was made using a biomass assay. In the majority of cases, treatment with AgNP caused a time and dose dependant degradation of chloroplasts and decrease in the photosynthetic activity of cells leading to the inhibition of aerial algae growth. However, some cases revealed an adaptive response of the cells. The response was induced by either a too low, or—after a short time—too high concentration of AgNP. Taken together, the data suggest that AgNP may be used as a biocide against aerial algal coatings; however, with a proper caution related to the concentration of the nanoparticles.

## Introduction

Progress in research on the effects of silver nanoparticles (AgNP) on biological systems has led to the application of these molecules to the public and commercial use [1, 2]. Experiments on different bacterial and fungal strains [3–8] have proven that AgNP, used either solely or as a compound combined i.e. with titanium dioxide, have a biocidal effect on heterotrophic microorganisms [9–17]. AgNP penetrate bacterial, fungal and animal cells [18, 19] and interfere with membrane proteins, activating a biochemical cascade that leads to an inhibition of cell division [20]. When passing through the cell wall and plasma membrane by diffusion or endocytosis, nanoparticles cause a mitochondrial dysfunction resulting in an increase of the reactive oxygen species (ROS). As a consequence, ROS cause oxidative stress and damage the surrounding proteins and nucleic acids [21]. The toxicity of AgNP depends highly on nanoparticle stability and mobility inside the biological systems and in the environment. Furthermore, the stability and mobility are the result of molecules`size, concentration, interactions with inorganic and organic compounds and many environmental parameters, i.e. temperature, pH, humidity. Thus, all these characteristic strongly influence the bioavailability and kinetics of AgNP molecules triggering various effects on cells [14, 18, 20, 22].

Algae are a highly diverse group of mainly photosynthetic microorganisms. Aquatic ecosystems are the most well-known algae environment. However, algae also colonize terrestrial habitats with extreme environments (deserts, glaciers). Such terrestrial species are known as aerophytic algae (=aerial algae) or airborne algae [23–25]. Aerial algae commonly colonize building constructions in all climate zones [26–28]. In the temperate zone, algal biofilms are formed mainly by unicellular and filamentous green algae (Chlorophyta) [28, 29]. They inhabit a variety of substrates such as wood, brick, concrete and grow in all seasons, both in high and low humidity conditions [30, 31]. These photosynthetic microorganisms yield invasive coatings that are extremely hazardous to the technical state of building materials. Aerial algal biofilms represent a biological factor in the degradation and deterioration of materials surface [28, 32, 33]. During many physiological and biochemical processes aerial algae release chemical compounds that erode building substrates and permit them to penetrate further into the material (especially hazardous in case of limestone substrates) [32, 34–41]. Algal biofilms degrade wood substrates, because algae periodically store water inside their cells and excrete it later, increasing dampness of the material surface. The spreading of aerial algae is a natural phenomenon induced by wind and weather conditions [24, 31]. Therefore, the measures taken against algal colonisation and thus, biodegradation of historic and utility structures, should include technological methods that allow for the eradication of existing biological contamination and inhibit their ability to re-colonize. Effective mitigation procedures involving the identification of degradation bio-factors and the analysis of cells sensitivity to biocides may have a positive effect on removal the facades coatings, what is particularly important in the case of conservation efforts aimed at preserving cultural heritage sites of a global importance [31, 33, 42–45].

The studies on algal response to AgNP exposure concern species such as: *Thalassiosira pseudonana* (marine diatom); *Synechococcus* sp. (freshwater cyanobacterium); *Chlorella vulgaris* and *Dunaliella tertiolecta* (freshwater and marine green algae respectively) or *Chlamydomonas reinhardtii* (green algae inhabiting small reservoirs or occurring on moist soil) [2, 14, 18, 22]; or include the assessment of algal coating growth inhibition on protected surface of underwater historical stones [46]. Mentioned studies however, were performed using aquatic species, the physiological processes of which are different from aerial ones [23, 47, 48]. The study on aerial algae response the biocidal properties of TiO_2_ nanometric compounds in combination with silver nanoparticles was provided by Goffredo and co-authors [41] and included cyanobacteria from the *Phormidium* and *Chlorogloeopsis* genera together with green algae from the *Klebsormidium* and *Chlorella* genera. So far there are no insights into how the AgNP treatment in isolation affects aerial algae cells. We set out to assess whether these molecules can be used as a biocide against aerial algal coatings for the protection and conservation of technical materials.

By using a model of aerial green algae–*Apatococcus lobatus*, we were able to assess the biocidal properties of AgNP against aerial algal biofilms causing the biodegradation and biodeterioration of building materials. This paper includes: (i) characterization of silver nanoparticles (ii) analyses of the changes in chloroplast structure and photosynthetic activity of *A*. *lobatus* cells under AgNP exposure basing on digital image analysis and chlorophyll fluorescence kinetic (iii) analysis of the growth inhibition rate under AgNP exposure based on the algae biomass assay (iv) assessment of the biocidal properties of AgNP against aerial algal biofilms.

## Material and methods

### Characterization of AgNP

Silver nanoparticles were provided by the tkNANO producer (Wroclaw, Poland) in the form of an aqueous dispersion in 8 ppm, 15 ppm, 20 ppm, and 107 ppm concentrations (74, 139, 185 and 992 μM/l, respectively). The nanoparticles were stabilized by polyelectrolytes and surfactants at a concentration of 0.15–0.5 g/l and 0.2–5 g/l respectively. The electrokinetic potentials were measured by means of electrophoretic light scattering, using Zetasizer 2000 apparatus (Malvern Instruments). Before the measurement, dispersions of nanoparticles were diluted in a citrate buffer, made of analytical grade citric acid (the final concentration was 7.5 mM), 1M sodium hydroxide and ASTM Type I deionized water; pH values were measured using a MM41 multimeter (Crison), equipped with a combination electrode. The concentration was kept constant at 27 ppm. The measurements were carried out at 25.0 ± 0.1°C, using the rapid mode. The value of Zeta potential reported for each sample corresponds to the average of five measurements.

Particle size measurements were performed using Photocor Complex (Photocor Instruments) apparatus, equipped with a 657 nm/28 mW laser and 288-channel autocorrelator, operating in multi-tau mode. Analysis of the dynamic light scattering (DLS) and Zeta potential were carried out in 14.8 mm round cells, submerged in decalin, as an index-matching liquid; the scattering angle was set at 90°, temperature of measurements 22.85 ± 0.05°C. The data analysis was performed using DynaLS v. 2.83 software (Alango Ltd.).

### Aerial algae culture

*Apatococcus lobatus* (Chodat) Petersen is an unicellular, coccoid green algae. It forms characteristic packets composed of two or more cells during its growth and development [49]. This species was chosen as a model for the study due to its broad ecological preference to environmental factors that make this species widespread. Together with other green algae, including *Desmococcus olivaceus*, *Chlorella* and *Klebsormidium* species complexes, *A*. *lobatus* is considered a very common and frequent species in aerial biofilms coating different technical materials [31, 41, 48, 50–52].

**Fig 1:**
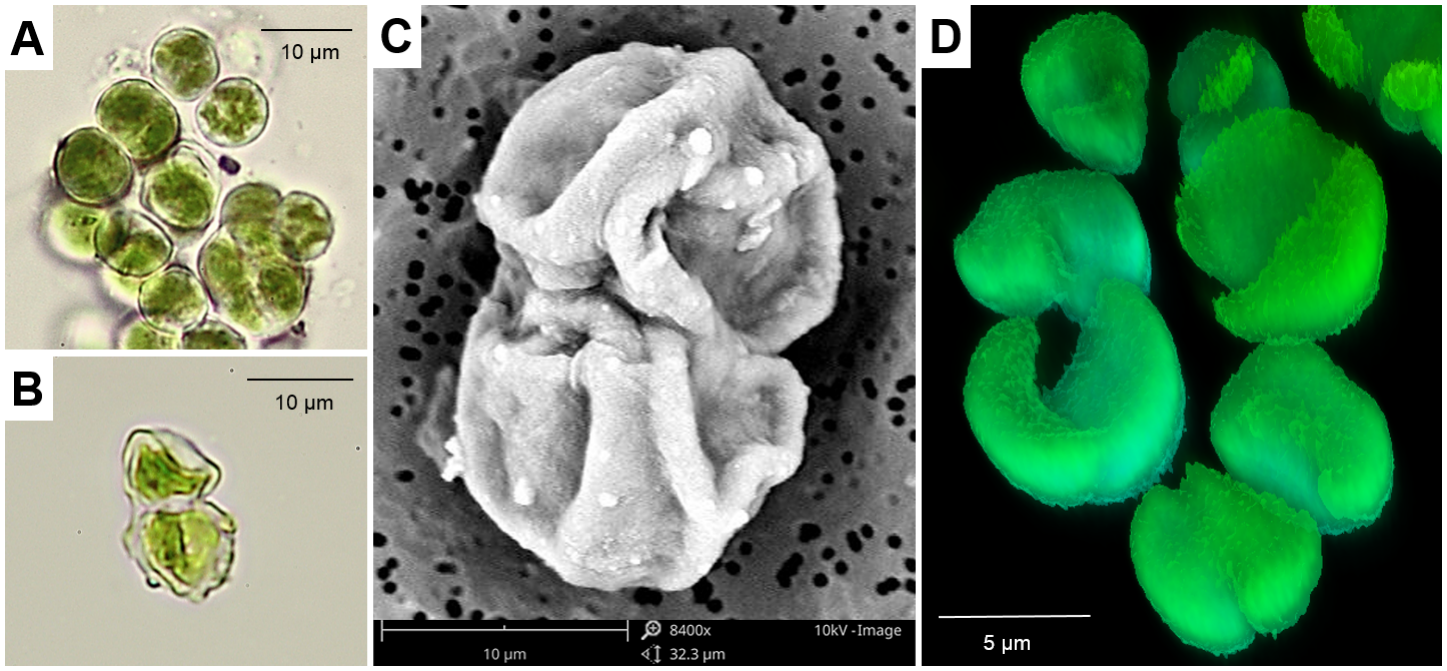
Cells of *Apatococcus lobatus* (strain PN003/1.1). a) cells growing in aqueous BBM (Light Microscope view); b) cells growing aerophytically on a BBM agar slant (Light Microscope view); c) cells growing aerophytically in SEM view; d) chloroplasts inside cells (3-D Confocal Laser Microscope view)

A single cell of *A*. *lobatus* was isolated from the wooden surface of a façade and germinated in the Laboratory of Algology and Mycology, University of Łódź (strain no. PN003/1.1, collected and isolated by P. Nowicka-Krawczyk) (Fig 1). Cultures were incubated on a BBM agar slant (Agar Difco^®^, v/v 1:1) [53] in optimal conditions (determined experimentally)–using artificial light of 22 μmolm^-2^s^-1^ with 16 hours day and 8 hours night periods; temperature of 20°C day and 15°C night (± 0.5°C); and relative humidity 50% (± 5%).

The vitality and condition of the strain were monitored while culturing using the NIKON Eclipse 50i Light Microscope (LM), the Phenom Pro-X Scanning Electron Microscope (SEM) and the Leica TCS SP8 Confocal Laser Microscope (CLM) in the Laboratory of Microscopic Imaging and Specialized Biological Techniques, University of Łódź.

### Exposure of *A*. *lobatus* to AgNP

Each sample was prepared using 2 cm^2^ of *A*. *lobatus* biofilm removed from the agar slant with a porcelain spatula and suspended in 2 ml of distilled water. The cells were spread over a 55 mm Petri dish (plate) with Agar Difco^®^ devoid of growing medium (to avoid interactions with medium compounds reducing AgNP toxicity) by gentle stirring. The plates were left under sterile conditions for water evaporation and placed in an incubator under the optimal conditions, previously mentioned. The cells were cultured continuously for six weeks and then treated with AgNP.

The AgNP concentrations in a volume of 3 ml were applied on plates using a pipette, keeping a part of plates without treatment (control cells). The reaction was examined at one hour (1 h), one day (24 h), one week (7 d) and two weeks (14 d) after exposure [14, 54–56]. To analyse the photosynthetic activity, cells from the central part of agar plates were taken using a 1.55 mm diameter micro-capillary, while the biomass assay was performed using the remaining biofilm.

The morphological changes in the structure of chloroplasts in cells under AgNP exposure were examined and analysed using a Leica TCS SP8 Confocal Laser Microscope with the LAS-AF 3.3.0.10134 software, and a NIKON Eclipse 50i light microscope equipped with CANON EOS digital camera.

### Analysis of the photosynthetic activity of *A*. *lobatus* under AgNP exposure

The photosynthetic activity analysis was based on the chlorophyll fluorescence intensity (^chl^FI) measurements. The measurements were made using a Leica TCS SP8 Confocal Laser Microscope with the LAS-AF 3.3.0.10134 software. The chlorophyll fluorescence excitation was induced using the White Light Laser (WLL) of 488 nm, while the detection was recorded in the PMT 3 channel at a 620–670 nm wavelength. Each sample was recorded/scanned in the ‘xyz’ axes to a depth of 40 μm (3-D scan). A depth in the ‘z’ axis reflecting a highest fluorescence (maximum pixel intensity) of cells was chosen for the measurements of ^chl^FI. For each sample, 28 measurements were performed in a spectrum length of 4.2 μm. The distribution of data was tested using the Kolmogorov-Smirnov test for normality. Since the data had non normal distribution and were independent, to find the statistical significance of ^chl^FI in cells treated with AgNP in relation to the control, the Kruskal-Wallis one-way analysis of variance by ranks (ANOVA K-W) supported with POST-HOC (Dunn Bonferroni) was used. The statistical analyses were performed with PQStat v. 1.6.2 software, with a significance level <0.05.

The changes in the photosynthetic activity of *A*. *lobatus* cells were estimated using the photosynthetic activity inhibition ratio (RIA^ph^) in the following expression: (^chl^FI_0_—chlFI_x_) / ^chl^FI_0_, where ^chl^FI_0_ is the chlorophyll fluorescence intensity of control cells and ^chl^FI_x_ the intensity of cells followed by *x* hours/days under AgNP exposure. The RIA^ph^ values range from 0 to 1, where 1 is a maximum level of photosynthetic activity inhibition.

### Analysis of the growth inhibition rate of *A*. *lobatus* under AgNP exposure

To analyse the growth inhibition rate (RI^g^) a biomass assay was performed using the measurements of chlorophyll *a* (chl *a*) concentration [23, 57, 58]. Chl *a* was extracted by homogenization from algal cells with 100% methanol. The suspension was left in the dark for 24 hours and then filtered through Millipore^®^ membranes (0.22 μm) under 3.7 PSI. The filtrate was kept in a dark test-tube for the absorbance (A) measurement. Five measurements of the absorbance were made for each sample, using the Spectroquanto Pharo 100 spectrophotometer at the 663 nm and 645 nm wavelength with methanol as a calibrator.

The biomass of cells (B) was calculated based on the average A and the equation: B = 12.7 A(663)− 2.7 A_(645)_ [23, 59, 60]. The distribution of data was tested using the Shapiro-Wilk test for normality. Since the data had a normal distribution and were independent with equal variances, the statistical significance of the biomass assay results in samples treated with AgNP in relation to the control was tested using the one-way analysis of variance (ANOVA), supported with POST-HOC (Fisher LSD). The statistical analyses were performed with PQStat v. 1.6.2 software with significance level <0.05.

The RI^g^ of cells under AgNP exposure was calculated using the expression: (B_0_—B_x_) / B_0_, where B_0_ was the biomass of control cells and B_x_ the biomass of cells followed by *x* hours/days after AgNP exposure. The assessment of the biocidal properties of AgNP against aerial algal biofilms was expressed as the percentage of RI^g^ after 14 days under AgNP exposure. The biocidal effect was considered as low (0 < %RI^g^ ≤ 33.3); medium (33.3 < %RI^g^ ≤ 66.6); and high (66.6 < %RI^g^ ≤ 100).

## Results and discussion

### Characterization of AgNP

**Fig 2:**
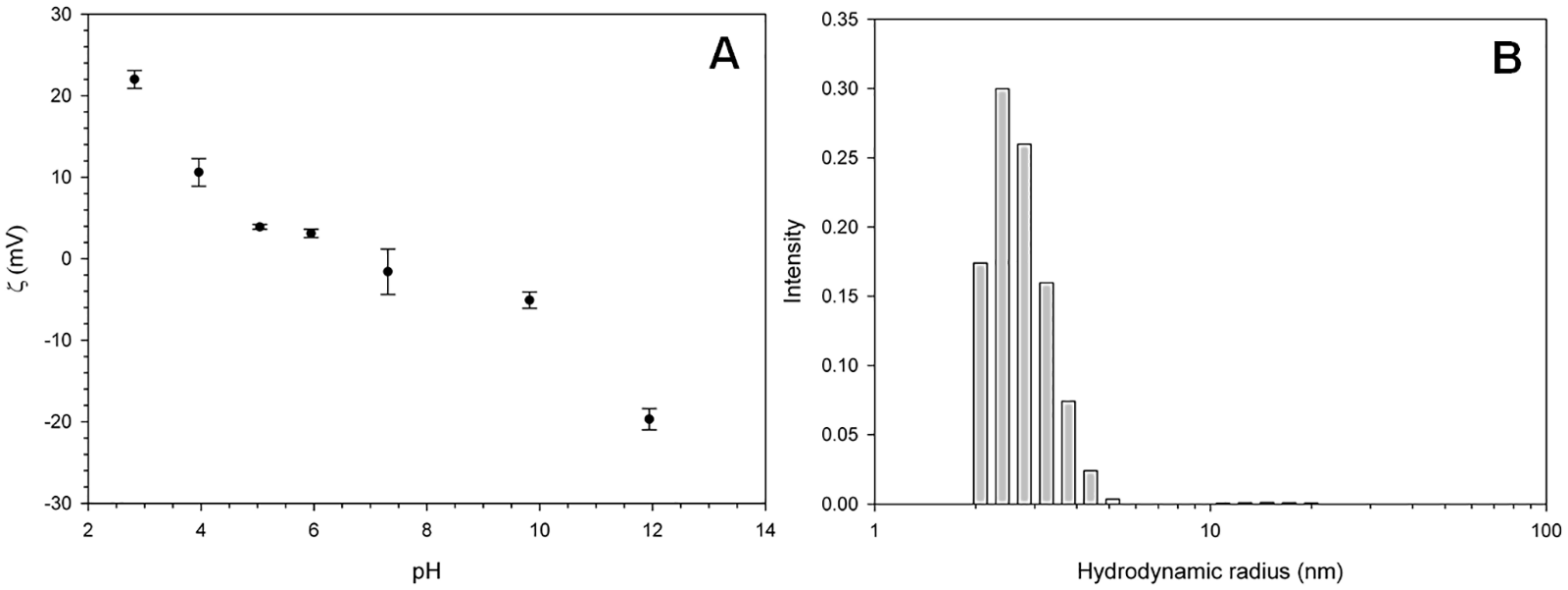
Electrodynamic and hydrodynamic characteristics of AgNP. a) Zeta potential of AgNP at different pH; b) DLS of AgNP.

The AgNP delivered by tkNANO in all concentrations were monodispersed and characterized by a spherical structure. The Zeta potential of AgNP ranged between 4.0 and 5.0 mV at the pH value ranging from 5 to 10 (Fig 2a). The average nanoparticles’ size showed by a dynamic light scattering analysis (DLS) was 3 nm (Fig 2b), while the mobility of 0.192 μmcm/V.s. All of the measured values were in accordance with a stable electrostatically stabilized nanoparticle dispersion.

In the case of spherical nanoparticles, the relationship between the specific surface area (SSA)—a property of molecules important for adsorption, catalysis and surface reaction, and their size, dictates that smaller particles have a higher SSA [18]. Studies on aquatic green algae revealed a clear relationship between nanoparticles size, associated with their SSA and the toxic properties [61]. Smaller particles have a greater ability to pass through the cell wall and plasma membrane during endocytosis and they can be easily transported within the cytosol [18]. Therefore, spherical AgNP with the size of 3 nm have a high ability to penetrate cells causing the toxic effect across cells, was thus chosen for the experiment.

### Analysis of photosynthetic activity of *A*. *lobatus* under AgNP exposure

**Fig 3:**
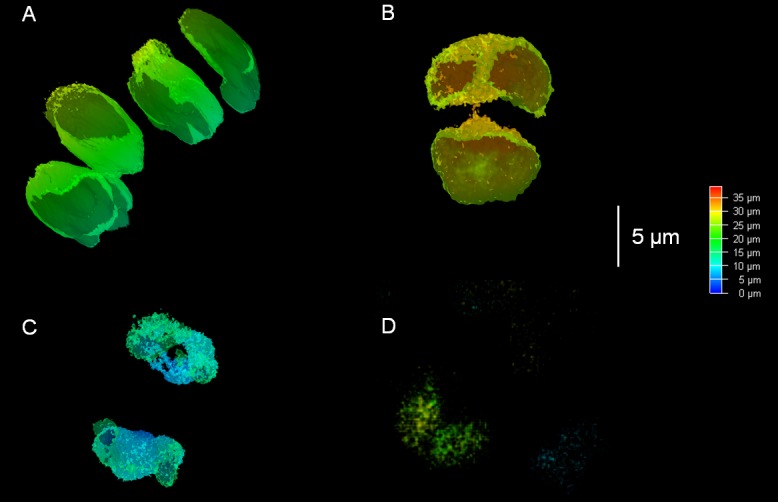
Digital imaging of the changes in *A*. *lobatus* chloroplasts under AgNP exposure (20 ppm); cross-sections in 3-D were performed using the Leica TCS SP8 Confocal Laser Microscope and the LAS-AF 3.3.0.10134 software. a) control samples; b) chloroplasts after 1 hour of AgNP exposure; c) chloroplasts after 24 hours of AgNP exposure; d) chloroplasts after 1 week of AgNP exposure.

The AgNP affected the cells of *A*. *lobatus* leading to the degradation of chloroplasts (S1 Fig). Initially, changes in the structure of chloroplasts affected their surface, which became more rough and uneven. After a day the chloroplasts collapsed and began to gradually degrade (Fig 3). After 2 weeks of exposure, in case of 20 ppm and 107 ppm AgNP no chloroplasts were visible in the CLM anymore.

The ^chl^FI of control cells increased up to 65% in relation to the 1^st^ hour of the experiment (Table 1). This was expected due to the optimal growth conditions at which the samples were cultured and it confirms the right conditions of the experiment. In all cases during the experiment, the ^chl^FI of cells treated with AgNP was lower than of the control cells. The differences between ^chl^FI of treated and control cells were statistically significant at the 1^st^ and 24^th^ hours of exposure (p<0.05), in the case of 15 ppm AgNP *p*-value was higher, but still significant. After a week, the differences of ^chl^FI of cells treated with 8 ppm AgNP and the control cells become statistically insignificant (p>0.05). Detailed results are presented in S1 Table.

**Table 1:** The photosynthetic activity inhibition ratio (RIA^ph^) and the growth inhibition ratio (RI^g^) of the *A*. *lobatus* cells treated with AgNP concentrations at every hour/day of the experiment and the concurrent chlorophyll fluorescence intensity (^chl^FI) and biomass (B) of the control cells; the statistical significance at the 0.05 level.

		AgNP exposure time				Aver.
		1 h	24 h	7d	14d	
^**chl**^**FI**	**Control**	89.91	97.73	112.65	148.00	112.07
**RIA**^**ph**^	**8 ppm**	0.096	0.552	0.463^a^	0.281^a^	0.348
	**15 ppm**	0.647	0.661	0.880	0.527	0.679
	**20 ppm**	0.784	0.413	0.743	0.978	0.730
	**107 ppm**	0.349	0.452	0.982	0.986	0.692
**B**	**Control**	3.536	3.564	3.790	4.051	3.735
**RI**^**g**^	**8 ppm**	0.127	0.338	0.289	0.291	0.261
	**15 ppm**	0.511	0.487	0.702	0.551	0.563
	**20 ppm**	0.593	0.400	0.646	0.906	0.636
	**107 ppm**	0.296	0.417	0.979	0.980	0.668

^a^ statistically insignificant, *p*-value > 0.05

**Fig 4:**
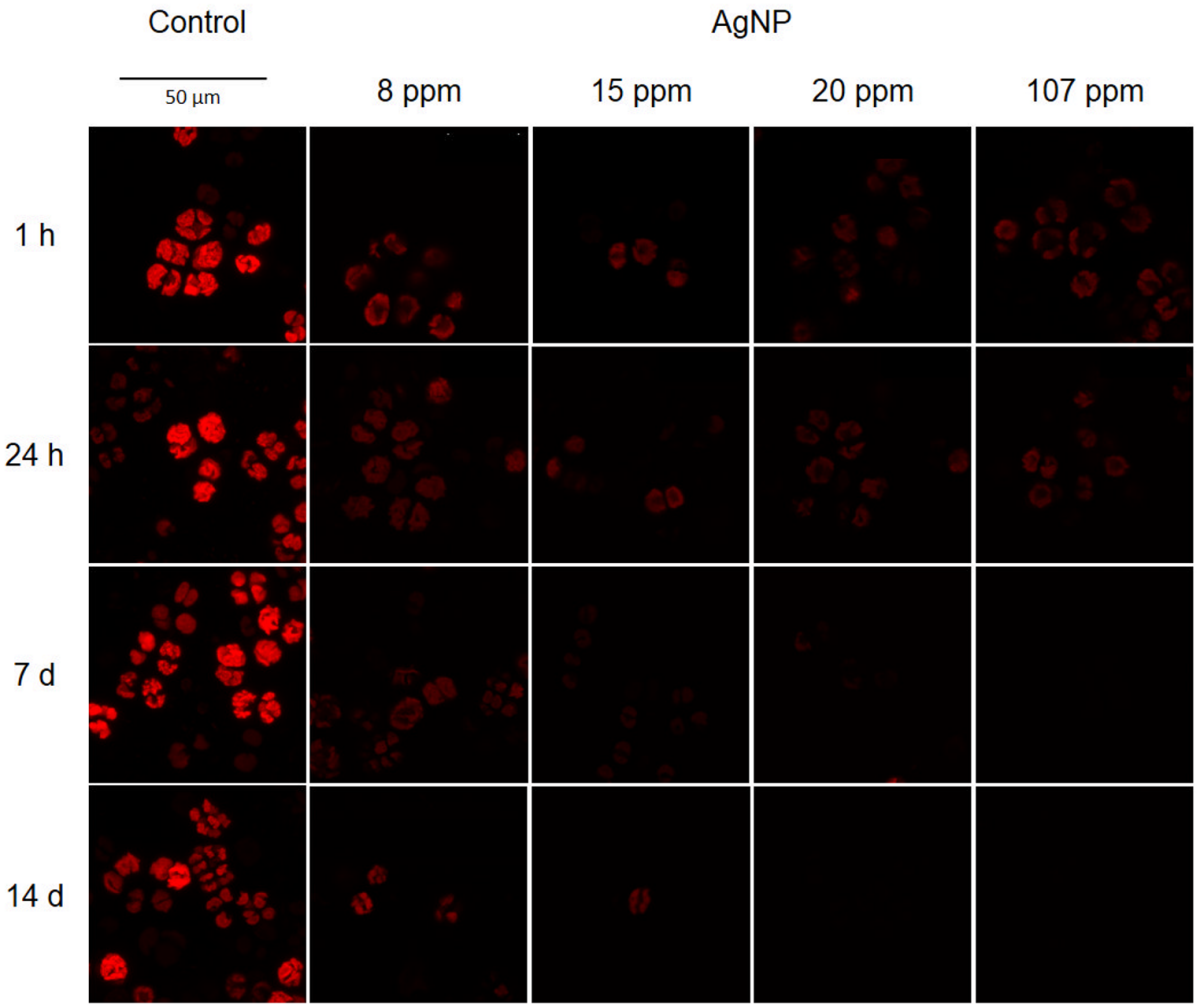
Extinction of chlorophyll fluorescence emission in the *A*. *lobatus* cells treated with AgNP concentrations. The digital images were captured every hour/day of the experiment using the Leica TCS SP8 Confocal Laser Microscope.

The deceleration of the photosynthetic activity ratio was dependent on dose and time. Under 8 ppm exposure, the highest RIA^ph^ was at the 24^th^ hour and it began decreasing with time. In the case of 15 ppm the inhibition of photosynthetic activity was observed until the 1^st^ week and then it became weaker—at the 14^th^ day the RIA^ph^ was lower than at the first hour of the experiment (Table 1). Treatment with 20 ppm caused a decrease of the photosynthetic activity of cells at the 1^st^ hour of exposure; however, during the first day of experiment this effect was reversed—the RIA^ph^ after the 24^th^ hour was lower by 47% in relation to the 1^st^ hour. The inhibition of the photosynthetic activity was noted and visible as the extinction of fluorescence emission only after the lapse of 24 hours (Fig 4). The RIA^ph^ at the 14^th^ day of the experiment was close to maximum (Table 1).

Surprisingly, in the case of 107 ppm—at the 1^st^ hour under exposure the photosynthetic activity of cells was higher than in the case of 15 and 20 ppm, thus the inhibition of photosynthesis was lower. Nevertheless, after 24 hours of exposure, this concentration caused a decrease in the photosynthetic activity of cells—the RIA^ph^ approached the maximum value at the 7^th^ day of exposure. While comparing the average RIA^ph^ the most effective inhibition of photosynthetic activity of aerial algal cells was 20 ppm AgNP (Table 1).

**Fig 5:**
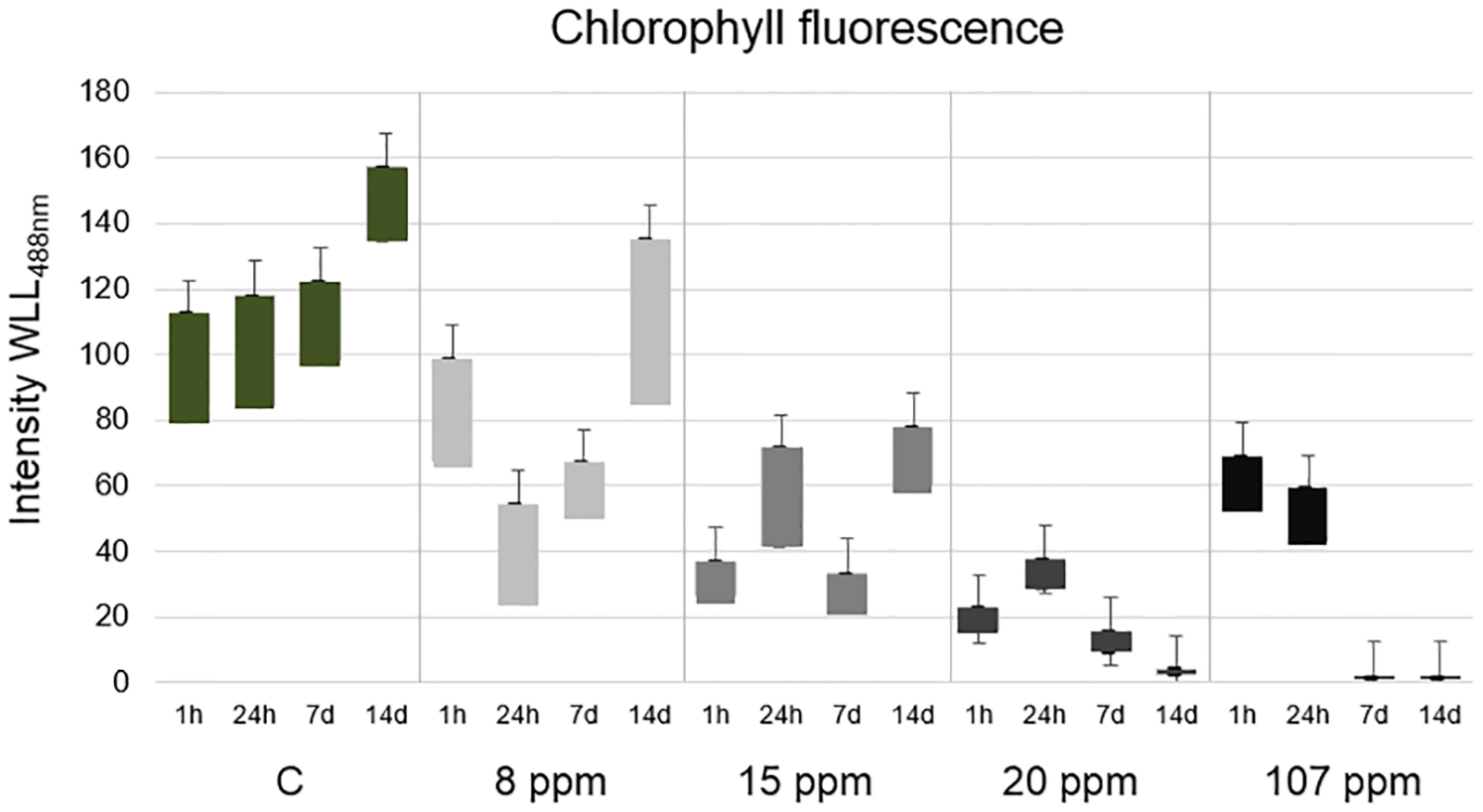
The range of chlorophyll fluorescence intensity of *A*. *lobatus* cells under AgNP exposure; the fluorescence was measured using the Leica TCS SP8 Confocal Laser Microscope equipped with WLL of 488 nm.

The chlorophyll fluorescence spectrum in each sample was diverse. The range of ^chl^FI is presented in Fig 5, while the detailed changes of spectrum are presented in S2 Fig. The lowest range of fluorescence emission was under the 20 ppm exposure, while the highest under 8 ppm. A high range of chlorophyll fluorescence emission may be evidence of an adaptive response of cells, which are attempting to counteract the toxic influence of nanoparticles. At lower concentrations, this antitoxic response is seemingly adequate for the recovery. Pillai and co-authors [62] observed increasing photosynthetic activity at the 1^st^ hour under 20 nM AgNP exposure that was related to the detoxifying response and the removal of silver ions from the cells. Higher concentrations overwhelm the adaptive response, which leads to toxicity and can be observed in the case of 20 ppm—during the first day of the experiment photosynthetic activity in cells treated with AgNP increased, but after that at the 14^th^ day it was fully inhibited. Furthermore, excessively high concentrations may also cause a strong detoxifying effect, which was observed in the case of 107 ppm AgNP. The photosynthetic activity of the *A*. *lobatus* cells treated with 107 ppm at the 1^st^ and the 24^th^ hour were higher than in the case of 15 and 20 ppm. This is likely related to the early adaptive response of cells. Algal cells can employ defence mechanisms to stop the metal ions before they penetrate the cell wall and plasma membrane. They can create specific chemical bindings to immobilize metals and make changes in the structure of cell wall and plasma membrane which block the cellular uptake [63].

The analysis of chlorophyll fluorescence emission provided detailed information on the changes of photosynthetic activity of cells under AgNP exposure. This method is often used in toxicological experiments on microalgae, since it allows minor changes in the chlorophyll fluorescence kinetic to be detected [2, 22]. The AgNP caused a noticeable decrease in the photosynthetic activity of the aerophytic algal cells. It is highly possible that this inhibition was related to a change in the maximum quantum yield for primary photochemistry and electron transport activity, as presented by Oukarroum and co-authors [22].

### Analysis of the growth rate inhibition of *A*. *lobatus* under AgNP exposure

**Fig 6:**
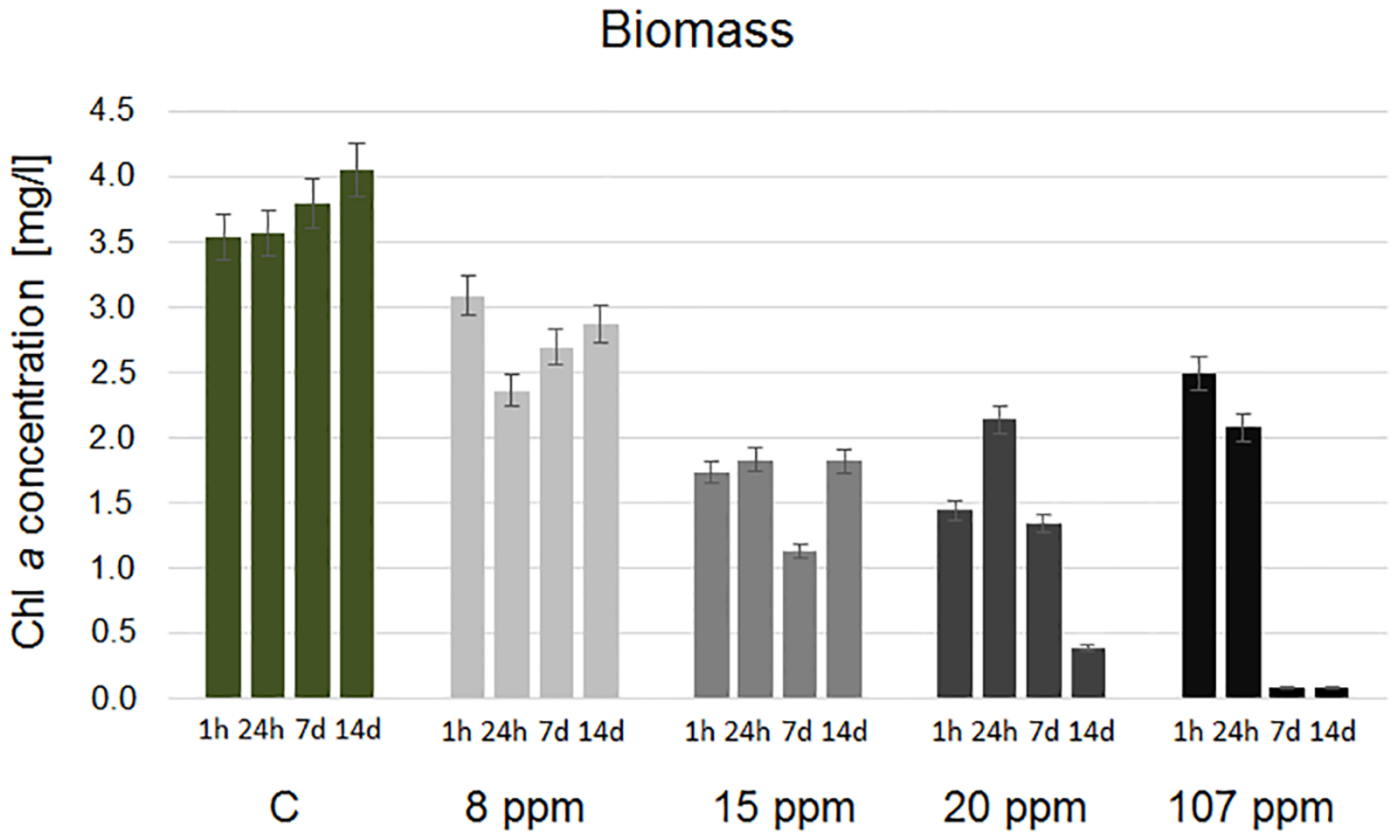
The biomass of *A*. *lobatus* cells under AgNP exposure; the biomass was expressed using chl *a* concentration.

The application of AgNP aqueous dispersion on *A*. *lobatus* biofilms resulted in the reduction of the cells biomass. The differences between the biomass of cells treated with AgNP and the control cells were statistically significant for all concentrations of nanoparticles (Table 1). The average biomass of control samples was 3.7 mg/l chl *a*. Under AgNP exposure, the biomass was reduced by 26% for 8 ppm, 56% for 15 ppm, 65% and 68% for 20 and 107 ppm respectively (Fig 6). The differences between particular samples were less noticeable than in the case of the photosynthetic activity analysis; nevertheless, the trend of changes in time confirmed, to a large extent, the results from previous analysis. The concentration of chl *a* in cells increased after the first day of the experiment under 8 ppm treatment (Fig 6). Furthermore, the concentration of chl *a* was quite similar in cells under 15 and 20 ppm exposure, but only for 20 ppm after the first day of the experiment (when the peak of concentration was recorded) it started to decrease. A stepwise degradation of chl *a* was noticed exclusively in cells treated with 107 ppm AgNP in the case of which the photosynthetic pigment was entirely degraded after a week (Fig 6).

Previous research shows that the aquatic algae exposure to AgNP results in a growth inhibition after 24 hours; however, the response is different depending on the algae group—cyanobacteria are more vulnerable than diatoms, which decrease their growth only at a concentration of 10 μM and higher [14]. In an aquatic environment, stable nanoparticles often occur in the form of a colloidal suspension or they aggregate into larger particles, which are mainly dependant on water temperature, pH and ion strength [18, 64]. The stability of nanoparticles fosters their mobility and resistance in the environment, therefore it is highly possible that aquatic algae are subjected to virtually constant interactions of nanoparticles. In the terrestrial environment the concentration of these molecules depends on the stationary and mobile sources, and any environmental or meteorological factors, such as: temperature, relative humidity, air turbulence, which may increase their condensation [65]. Furthermore, their concentration usually decreases exponentially with downwind distance from their sources [66]. The different behaviour of nanoparticles in the atmosphere and aquatic environment leads to the altered vulnerability of aerophytic and aquatic algal cells. Moreover, biochemical processes in aerophytic algal cells are adapted to the extreme “*land*” environment i.e. high insolation, low humidity and low accessibility to the nutrient compounds [28, 47, 52]. As a consequence, both the behaviour of molecules and cells vulnerability may result in a variable toxic effect of AgNP on aquatic and aerophytic algal cells. To assess the biocidal properties of AgNP on terrestrial biofilms and their usefulness as a tool against biodegradation and biodeterioration of building materials, it is necessary to use a proper biological factor of degradation for an experiment—in this case the most frequent and widespread aerophytic green algae was used.

All of the tested concentrations of AgNP inhibited the biological activity (photosynthetic activity and biomass) of the aerophytic algae, but the inhibition had a time-dependent manner and in some cases it was reversed after 2 weeks of the experiment. The strongest impact was revealed under 107 ppm exposure (Table 2). The aqueous dispersion of AgNP in such concentration has a high biocidal effect on aerial photosynthetic biofilms. Nevertheless, a positive reaction was also observed in the case of 20 ppm dispersion, but the effect was extended in time and it reached a high level between the first and the second week. The medium and low biocidal effect was noted for 15 ppm and 8 ppm dispersions respectively; furthermore, their biocidal properties appeared to decrease with time (Table 2). Thus, AgNP can be useful as a tool against aerophytic algal coatings, however only at a proper concentration.

**Table 2:** Percentage of the growing inhibition rate and the biocidal effect of AgNP against aerophytic algal cells after 14 days of AgNP exposure.

	AgNP exposure			
	8 ppm	15 ppm	20 ppm	107 ppm
**1 h**	12.7	33.8	28.9	29.1
**24 h**	51.1	48.7	70.2	55.1
**7 d**	59.3	40.0	64.6	90.6
**14 d**	29.6	41.7	97.9	98.0
**Biocidal effect**	**Low**	**Medium**	**High**	**High**

## Supporting information

S1 Fig Morphological changes of the chloroplasts in *A*. *lobatus* cells under AgNP exposure. A-B) Control cells with a properly formed chloroplast; C-G) following stages of chloroplasts degradation; H-I) dead cells without chloroplast.
(PDF)

S2 Fig Changes of the chlorophyll fluorescence spectrum of the *A*. *lobatus* control cells and cells treated with AgNP concentrations at every hour/day of the experiment. (PDF)

S1 Table The statistical significance (p-level <0.05) of the chlorophyll fluorescence intensity and biomass of *A*. *lobatus* cells treated with AgNP concentrations at every hour/day of the experiment. (PDF)
