# Supplementary material for: Silver nanoparticles as a control agent against facades coated by aerial algae—A model study of Apatococcus lobatus (green algae)
Source: PLoS One. 2017 Aug 14;12(8):e0183276. doi: 10.1371/journal.pone.0183276 (PMC5555565; doi:10.1371/journal.pone.0183276)
Supplement: S1 Fig — A-B) Control cells with a properly formed chloroplast; C-G) following stages of chloroplasts degradation; H-I) dead cells without chloroplast. (PDF) [file pone.0183276.s001.pdf]

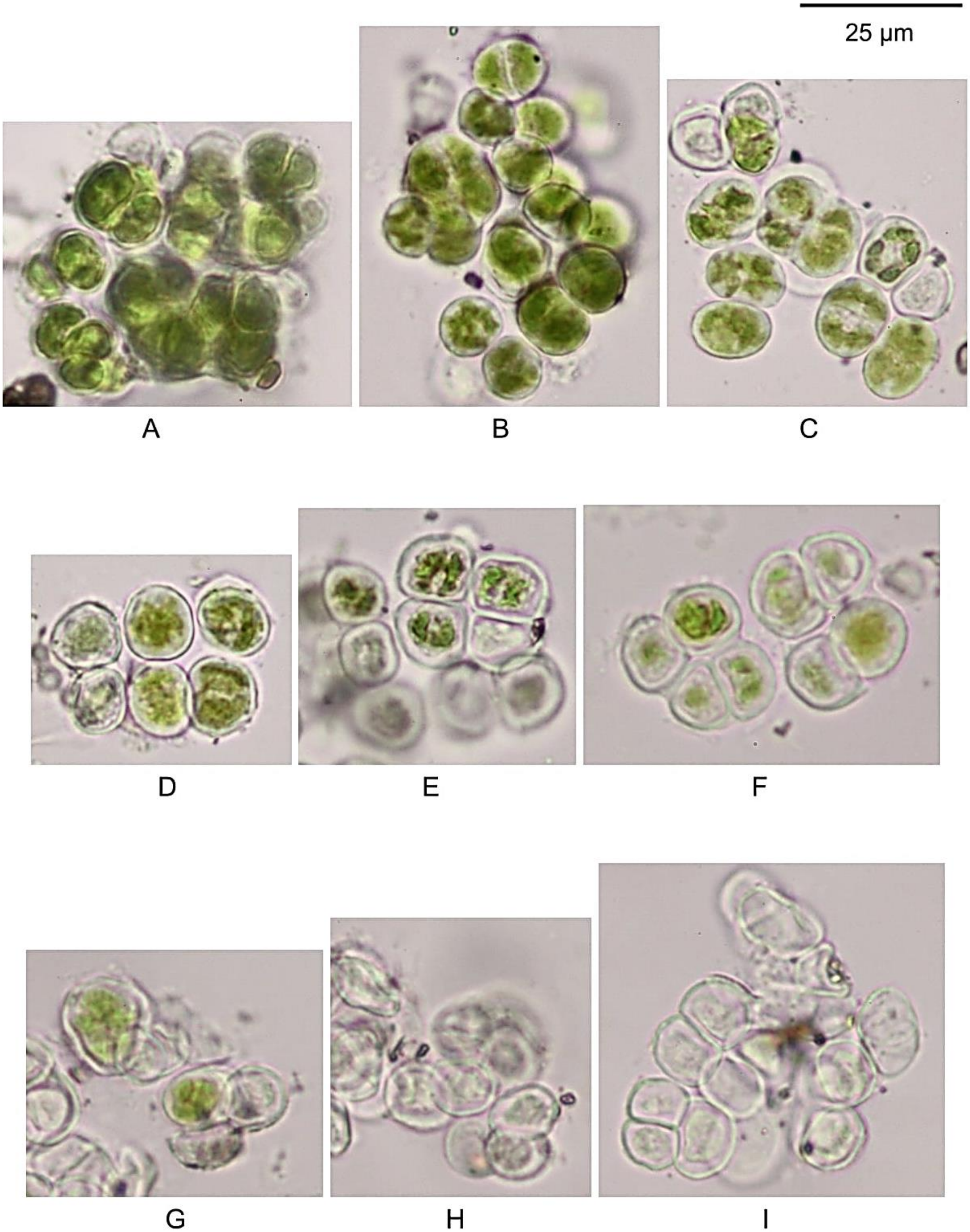

S1 Fig. Morphological changes of the chloroplasts in *A. lobatus* cells under AgNP exposure. A-B) Control cells with a properly formed chloroplast; C-G) following stages of chloroplast degradation; H-I) dead cells without chloroplast.
