# Supplementary material for: Silver nanoparticles as a control agent against facades coated by aerial algae—A model study of Apatococcus lobatus (green algae)
Source: PLoS One. 2017 Aug 14;12(8):e0183276. doi: 10.1371/journal.pone.0183276 (PMC5555565; doi:10.1371/journal.pone.0183276)
Supplement: S2 Fig — (PDF) [file pone.0183276.s002.pdf]

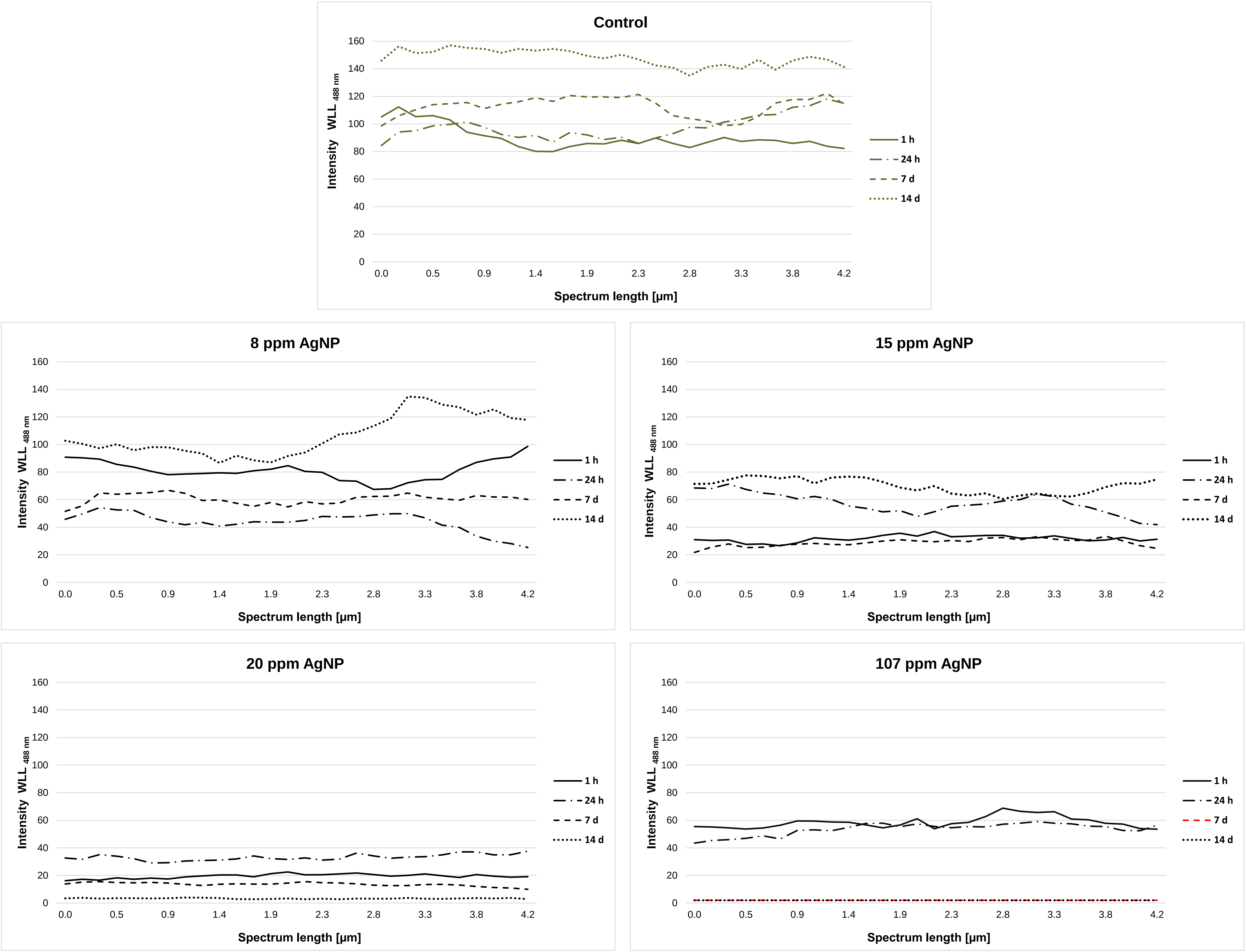

S2 Fig. Changes of the chlorophyll fluorescence spectrum of the *A. lobatus* control cells and cells treated with AgNP concentrations at every hour/day of the experiment.
