# Supplementary material for: Silver nanoparticles as a control agent against facades coated by aerial algae—A model study of Apatococcus lobatus (green algae)
Source: PLoS One. 2017 Aug 14;12(8):e0183276. doi: 10.1371/journal.pone.0183276 (PMC5555565; doi:10.1371/journal.pone.0183276)
Supplement: S1 Table — (PDF) [file pone.0183276.s003.pdf]

S1 Table. The statistical significance (*p*-level <0.05) of the chlorophyll fluorescence intensity and biomass of *A. lobatus* cells treated with AgNP concentrations at every hour/day of the experiment.

		^{chl}FI under AgNP exposure[a]				B under AgNP exposure[b]			
		**8 ppm**	**15 ppm**	**20 ppm**	**107 ppm**	**8 ppm**	**15 ppm**	**20 ppm**	**107 ppm**
**Control**	**1 h**	$<1\times10^{-6}$	0.002	$<1\times10^{-6}$	$5,9\times10^{-5}$	$<1\times10^{-6}$	$<1\times10^{-6}$	$<1\times10^{-6}$	$<1\times10^{-6}$
	**24 h**	$<1\times10^{-6}$	0.002	$<1\times10^{-6}$	$5,9\times10^{-5}$	$<1\times10^{-6}$	$<1\times10^{-6}$	$<1\times10^{-6}$	$<1\times10^{-6}$
	**7 d**	0.095	2×10^{-6}	$<1\times10^{-6}$	$<1\times10^{-6}$	$<1\times10^{-6}$	$<1\times10^{-6}$	$<1\times10^{-6}$	$<1\times10^{-6}$
	**14 d**	0.095	2×10^{-6}	$<1\times10^{-6}$	$<1\times10^{-6}$	$<1\times10^{-6}$	$<1\times10^{-6}$	$<1\times10^{-6}$	$<1\times10^{-6}$

[a] *p*-value of ANOVA K-W with POST-HOC – Dunn Bonferroni

[b] *p*-value of ANOVA with POST-HOC – Fisher LSD
